# The Role of Interleukin-6 in Bone

**DOI:** 10.1210/jendso/bvaa112

**Published:** 2020-09-07

**Authors:** Joseph A Lorenzo

**Affiliations:** 1 Department of Medicine, UConn Health, Farmington, Connecticut; 2 Department of Orthopedics, UConn Health, Farmington, Connecticut

**Keywords:** interleukin-6, osteoclasts, bone resorption

Interleukin 6 (IL-6) was first identified in 1973 as a T cell secreted factor, which regulated antibody production by B cells. Since then, it has been shown to be a proinflammatory cytokine with a wide variety of activities in multiple cell types, including those in bone [1]. Its receptor can be either membrane bound on cells producing “cis” signaling or soluble producing “trans” signaling [1]. Although it is often thought of as proresorptive, its effects on osteoclasts and bone mass in animal models have been variable [2]. This discrepancy may be related, in part, to differences in the responses of bone cells to membrane-bound and soluble-bound IL-6 receptor signaling [3]. Global deletion of IL-6 in mice results in a phenotype of increased bone mass [4]. In humans, serum levels of IL-6 predict bone loss during the first decade after the menopause [5] and polymorphisms of the *IL6* gene associate with bone mineral density [6]. Hence, there is evidence that IL-6 can influence bone cells and ultimately bone mass, but its effects on bone appear multifaceted and, particularly for human physiology, not completely understood.

In the current issue of the *Journal of the Endocrine Society*, Lehrskov et al [7] examines the effects that tocilizumab or infusion of IL-6 had on serum markers of bone turnover. Tocilizumab is a neutralizing monoclonal antibody to human IL-6 receptor that blocks signaling mediated by both the soluble- and membrane-bound IL-6 receptor. Three studies were analyzed.

Study 1 was a single-blind, crossover study consisting of a 1-hour infusion of saline (placebo) and 7 days later of tocilizumab, followed by a bout of exercise and a mixed meal tolerance test (MMTT). Blood was drawn for analysis at 20-minute intervals during exercise, which was followed by an MMTT with blood drawn, subsequently, for 3 hours. Five subjects were analyzed.

Study 2 was a randomized, double-blind 12-week exercise training intervention study in which subjects received infusions of saline or tocilizumab throughout the study period. Subjects in study 2 were randomized into 4 blocks of 13 subjects each: (1) no exercise + placebo, (2) no exercise + tocilizumab, (3) exercise + placebo, and (4) exercise + tocilizumab. The exercise was aerobic, high-intensity interval training performed on an ergometer bike. At the conclusion of the study, subjects underwent an MMTT and, subsequently, blood was drawn at 30 minute intervals for 2 hours. Fifty two subjects participated in this study.

Study 3 was a randomized, double-blind, crossover study consisting of a 30-minute infusion of saline or IL-6 followed by an MMTT and subsequent blood draws for 2 hours. Ten subjects were analyzed. Studies 1 and 3 involved a small number of subjects, and none of the studies used intention to treat statistical analyses.

The main outcome of all studies was the effects that manipulation of IL-6 (either negatively with tocilizumab or positively with IL-6 infusion) had on 2 serum bone turnover markers: C-terminal telopeptide (CTX), a measure of bone resorption, and procollagen 1 intact N-terminal propeptide (P1NP), a measure of bone formation.

In all 3 studies the outcome was similar. The authors found no effect of IL-6 manipulation on either outcome measure, nor did tocilizumab alter bone mass at the conclusion of study 2. Of note, the authors remark in the discussion that “12 weeks of exercise training increased levels of osteocalcin and moreover that combining exercise training with IL-6 receptor blockade prevented exercise from increasing osteocalcin levels.” Hence, these data imply that there were effects of IL-6 signaling blockade on bone cells that were not evident if only CTX and P1NP were examined. As a conclusion, the authors state that “IL-6 may not regulate bone remodeling in humans.”

While it is premature to completely agree with this conclusion, the results do bring into question the concept that IL-6 has important effects on bone in healthy humans (ie, those not undergoing an inflammatory process). It is known that deletion of IL-6 in mice prevents the decrease in bone mass that is seen after ovariectomy or experimental inflammatory arthritis [1]. In addition, the reduced bone mass of IL-6 deficient mice is not evident at 4 months of age [8] but is at 8 months [4]. This result suggests that inflammation associated with aging may be involved. Recent evidence demonstrates that “trans” IL-6 signaling, involving extracellular binding of IL-6 and its soluble receptor is more important for the osteoclastogenic response to IL-6 in rheumatoid arthritis, estrogen deficiency, and colitis [1]. Hence, it is possible that healthy individuals lack sufficient soluble IL-6 receptor levels in the bone microenvironment to produce effects on bone from either the available IL-6 that normally exists there or from the infused IL-6 that was administered to healthy subjects in study 3 of Lehrskov et al [7]. In contrast, in states characterized by enhanced inflammation, soluble IL-6 receptor levels may be increased, allowing “trans” signaling of IL-6 in bone.

Clearly, additional work on this topic needs to be done in both animal models and humans. The studies of Lehrskov et al [7] in this issue of the *Journal of the Endocrine Society* are a start but they are preliminary. More comprehensive investigation of the effects of IL-6 blockade will, more importantly, further our understanding of the role of IL-6 in bone metabolism in states of health and disease.

## Additional Information

***Disclosure Summary:*** The author has nothing to disclose.
